# Efficient genome editing in dicot plants using calreticulin promoter-driven CRISPR/Cas system

**DOI:** 10.1186/s43897-024-00128-w

**Published:** 2025-02-02

**Authors:** Bingjie Li, Yun Shang, Lixianqiu Wang, Jing Lv, Qi Wu, Fengjiao Wang, Jiangtao Chao, Jingjing Mao, Anming Ding, Xinru Wu, Kaili Xue, Chen Chen, Mengmeng Cui, Yuhe Sun, Huawei Zhang, Changbo Dai

**Affiliations:** 1 Tobacco Research Institute, Chinese Academy of Agricultural Sciences, Qingdao, 266101 China; 2 State Key Laboratory of Wheat Improvement, Peking University Institute of Advanced Agricultural Sciences, Shandong Laboratory of Advanced Agricultural Sciences in Weifang, Weifang, Shandong 261325 China

Genome editing mediated by Clustered regularly interspaced short palindromic repeats (CRISPR)/Cas systems is widely used to study functional genomics and improve the agronomic traits in crops (Hua et al. 2019; Gao 2021). To date, various plant-specific CRISPR/Cas expression systems have been developed and applied in model plants, including Arabidopsis, rice, and tobacco, however, editing efficiencies vary widely (Ma et al. 2016; Tian et al. 2020). A prototypical CRISPR/Cas system with the *35S* promoter (*35Spro*) and *pol III* promoter can accomplish genome editing in plants, but generates relatively few homozygous/bi-allelic (ho/bi) mutations, especially in polyploid crops (Ma et al. 2016, 2023). CRISPR/Cas expression cassettes, especially promoters used for driving both Cas9 and sgRNAs, are largely correlated to editing efficiency in plants (Ma et al. 2023). Here, we report a novel efficient promoter that can be used to drive Cas9 nuclease and generate robust editing efficiency in T0 regenerated dicot plants.

Using proper promoters for CRISPR/Cas systems is critical for gene editing, so we presumed that promoters exhibiting high activities in tobacco calli might improve the genome editing efficiency in plants. We noticed that one of the plant callus high expression (*PCE*) genes, termed *PCE8*, which encodes a calreticulin-like protein (CRT), exhibits an extremely high expression level in the early stage of tobacco shoot regeneration (Fig. S1). Its promoter (*PCE8pro*) contains cis-elements related to meristem expression, hormone and wound response (Fig. S2). We speculated that the *PCE8pro* was suitable to produce efficient targeted editing in tobacco genome. To validate this hypothesis, we developed three types of CRISPR/Cas9 expression cassettes, pDC30, pDC40, and pDC45, in which the sgRNA is controlled by *U6-26* or *35Spro*, tRNA-sgRNA-EU unit (Xie et al. 2015; Diamos & Mason 2018), and *SpCas9* is independently controlled by the *35Spro* and *PCE8pro* (Fig. 1A).

**Fig. 1:**
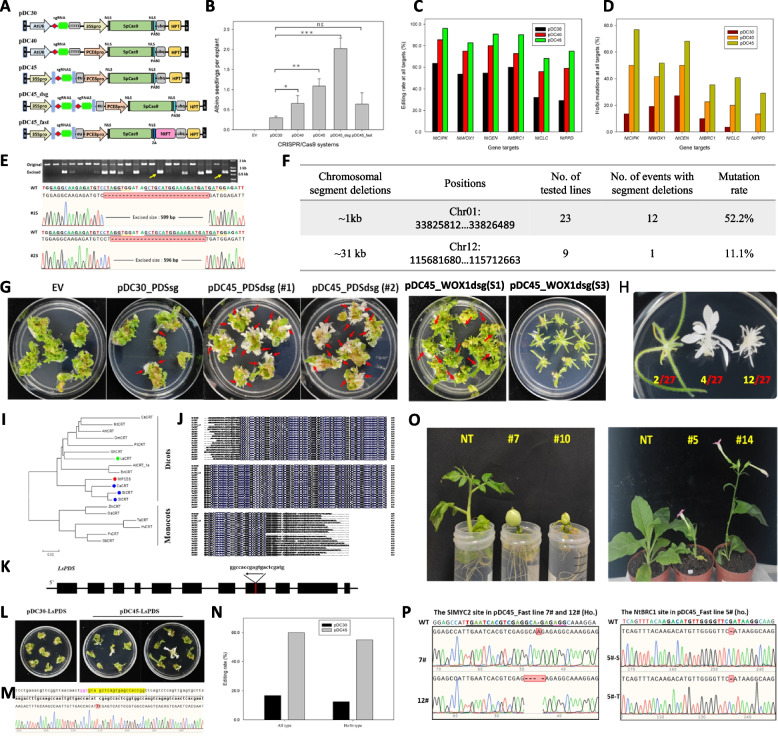
Efficient genome editing in dicots using calreticulin promoter-driven CRISPR/Cas system. **A** Schematic illustration of CRISPR/Cas9 systems used in this study. NLS: nuclear location signal; eu: intronless tobacco *extension* terminator; 2A: self-cleaving 2A peptide. The bar in light blue: tRNA sequence. **B** Comparison of the ho/bi mutations based on the ratios of albino plantlets generated by targeting tobacco *PDS* genes with various CRISPR/Cas9 systems, the standard deviation (SD) is derived from three replicates. **C** Comparative analysis of mutation editing efficiencies in the tobacco T0 generation generated via pDC30, pDC40, and pDC45 in the *NtCIPK, NtWOX1, NtBRC1, NtCEN, NtCLC* and *NtPPD* target genes. **D** The ho/bi mutations of tobacco T0 seedlings induced by various CRISPR/Cas9 cassettes for the six targeted genes in this study. **E** Agarose gel electrophoresis and Sanger sequencing of fragment deletions generated by pDC45_PDSdsg. The target was *NtPDS* gene. The yellow arrowheads show the excised fragments used for sequencing. **F** Chromosomal segment deletion efficiency created by the pDC45_dsg system. The location of the chromosomal fragment deletion is referenced to the tobacco reference genome. **G** The phenotype of transgenic tobacco lines generated by various pDC constructs. Plates #1 and #2 display randomly selected explants from two individual experiments. Red arrowheads indicate the absolute albino shoots of *NtPDS* disruption seedlings, as well as the bladeless leaf phenotype produced by the targeted knockout of *NtWOX1* allelic genes. “S1” and “S3” indicate different stages of tobacco regeneration. EV, pDC45 vector without a target sequence. **H** Diverse phenotypes of tobacco ho/bi T0 lines with *pDC45_PDSsg* + *WOX1sg* construct. The numbers highlighted in yellow and red show the count of ho/bi lines and Hyg-resistant lines, respectively. **I** Phylogenetic tree of CRT proteins based on amino acid sequences from different species. The sequence information employed for alignment is detailed in the supplementary materials. Monocots and dicots were indicated in different groups. A filled circle with red and blue was marked before tobacco PCE8 and other CRTs in *Solanum* species. The light green filled circle represents lettuce CRT protein. **J** Sequence alignment of CRTs in different plant species. Amino acid sequences were aligned using the DNAMAN alignment program. **K** Gene structure and target sites of lettuce *LsPDS* gene. **L** The phenotypes of T0 seedlings generated by pDC30 and pDC45 vector, The albino shoots indicate complete knockout of lettuce *PDS* gene. **M** Sanger sequencing of PCR product from *LsPDS* edited lines. Nucleotide sequences are indicated by yellow for the target and magenta for the PAM sequence, respectively. **N** Editing efficiency analysis of T0 transgenic lettuce harboring different editing systems. “All types” indicates that every form of editing events was included. “ho/bi type” is restricted to the enumeration of solely ho/bi mutations. **O** Observation of early bolting and fruit setting in T0 transgenic tomato and tobacco. ‘NT’ stands for non-transgenic lines while ‘#’ refers to individual edited lines. **P** Sequence chromatogram of PCR products of pDC45_Fast transgenic plants, the spacer and PAM sequence is underlined

To assess our systems, we selected tobacco *Phytoene desaturase* (*NtPDS)* as a target gene. Seedlings with disrupted *NtPDS* genes exhibit an albino phenotype, making it easy to determine whether both *NtPDS* alleles are edited in S- and T-subgenome (Gao et al. 2015). For T0 lines transformed by the pDC30 cassette, we yielded an average of 0.3 albino shoots per explant. Using the same strategy, we obtained 0.66, and 1.09 albino seedlings per explant on average from pDC40, and pDC45 transformed tobacco, which are respectively 2.2- and 3.6-fold higher than in the pDC30 construct (Fig. 1B). We further designed six gRNAs respectively targeting *Calcineurin B-like protein (CBL) interacting protein kinase (NtCIPK), WUSCHEL related homeobox 1-like (NtWOX1), BRANCHED 1 (NtBRC1), CENTRORADIALIS (NtCEN), Chloride channel (NtCLC) and PEAPOD (NtPPD)* in allotetraploid tobacco (Table S1). High-throughput sequencing results indicated that 56.0% to 90.0% of pDC40 transformed plants were mutated at all target sites, and 13.6% to 55.0% of lines displayed ho/bi mutations, while pDC30 construct resulted in a lower range of ho/bi mutations, varying from 0% to 22.7%, suggesting calreticulin promoter-driven Cas9 system is highly efficient in generating ho/bi targeted mutations in tobacco (Fig. 1C-D, Table S1). Additionally, the ho/bi mutations of pDC45 transgenic lines were further improved as compared to those achieved using the pDC30 or pDC40 constructs in tobacco (Fig. 1D). To investigate whether the enhanced editing efficiency was related to activity of the *PCE8pro*, we quantified *Cas9* gene expression in tobacco callus respectively transformed with pDC30, pDC40, or pDC45 constructs. As expected, *PCE8pro* resulted in higher *Cas9* expression levels than that driven by the 2X35S promoter (Fig. S3).

Since the deletion of chromosomal segments in plants plays a crucial role in the creation of novel plant germplasm (Duan et al. 2021), we developed pDC45 dual sgRNA (pDC45_dsg) systems to achieve multiplex genome editing in tobacco. Two gRNAs were designed to simultaneously target both *PDS* allelic genes in tobacco. We obtained a 6.73-fold increase in albino plants from pDC45_dsg transformed tobacco compared to the pDC30 construct (Fig. 1B, G, S4). Additionally, 52.2% of tested albino plants had fragment deletions from the two cleavage sites from pairs of *NtPDS* sgRNAs (Fig. 1E, F). The fragment deletion of pDC45_dsg transgenic lines were further confirmed through dual gRNAs-mediated targeting of tobacco *NtWOX1*. Notably, over 60.0% of the ho/bi transgenic lines exhibits visible excised fragments resulting in the production of bladeless leaves owing to disruption of both *NtWOX1* loci (Fig. 1G, S5). We then explored its potential to create large chromosomal segment deletions using a combination of specific gRNAs. As expected, we obtained a unique amplified product from nine transgenic lines, but not from wild-type, representing chromosomal segment deletion size of 30.9 kb and achieving a deletion efficiency of 11.1% (Fig. 1F, S6).

To further evaluate the multiplex genome editing capability of pDC45_dsg systems, we selected two reported gRNAs, separately targeted to tobacco *FAD2-2* and *CLC* alleles, with extremely low ho/bi mutations (11.1% and 2.4%), and simultaneously introduced them into tobacco using pDC45_dsg construct. Among 23 T0 plants, 65.2% and 34.8% of lines contained ho/bi mutations at *FAD2-2* and *CLC* sites, respectively, indicating that pDC45_dsg system improved editing activities at sites with poor sgRNA efficiency (Table S2). Moreover, we examined dual gRNAs simultaneously targeted to *NtBRC1* and *NtCCD8* genes, which are correlated to outgrowth of axillary buds in tobacco (Fig. S7). The editing efficiency was up to 100.0% at targeted sites except that in the *NtBRC1-T* locus, and the ho/bi mutations of T0 lines at four sites were 95.0%, 90.5%, 81.0%, and 38.1%; 33.3% of tested lines contained ho/bi mutations at all targeted sites (Table S2). We constructed another cassette, targeting *NtWOX1* and *NtPDS* using pDC45_dsg cassette. Among 27 T0 lines, 85.2% to 96.3% of them carried targeted mutations at each site, and 66.7% of tested plants were albino, bladeless, or albino bladeless leaves, due to complete disruption of all targeted alleles (Fig. 1H, Table S2).

Considering the high conservation of calreticulin-like protein in plants, we anticipate that PCE8pro-controlled systems may be effective in other dicots (Fig. 1I-J). To confirm our hypothesis, we evaluated the editing efficiency of the pDC45 expression system in lettuce (*Lactuca sativa*), a globally cultivated vegetable crop and another member of the dicotyledonous plants. The application of the pDC45 system to target the *LsPDS* gene led to a robust gene editing efficiency of 60.0%, with a notable 55.0% of plants displaying albino phenotypes, indicative of ho/bi mutations in lettuce. In contrast, the pDC30 vector was less effective, with a gene editing rate of merely 16.5% and only 12.5% of plants showing the desired albino traits (Fig. 1K-N). To evaluate the editing efficiency of the pDC45 vector in tomatoes, we targeted the powdery mildew resistance factor, Mildew Locus O *SlMLO* (Ma et al. 2023), and the JA signaling pathway basic helix-loop-helix (bHLH) master transcription factor, SlMYC2 (Guo et al. 2021) Upon introducing the *pDC45_MLOsg* and *pDC45_MYC2sg* + *MLOsg* constructs into tomato, we observed ho/bi mutation efficiencies of 73.9% and 45.5%, respectively, for single and double mutations in T0 tomato lines (Table S2), indicating that pDC45 systems enable engineering the gene mutations in both lettuce and tomato genome. To further investigate the conservation and versatility of the *PCE8* gene, we cloned the 1.5 kb upstream promoter region of the *PCE8* homolog in lettuce to construct the pDC46 editing vector. Examination of editing efficiency at the *PDS* gene in both lettuce and tobacco showed ho/bi proportions of 60.0% and 37.5%, respectively, thus demonstrating that the *LsPCE8* promoter could effectively facilitate genome editing in both species (Fig. S8, Table S3). These findings suggest that *PCE8* promoters derived from different species could provide high-efficiency genome editing across dicot species, highlighting the generalizability of this promoter.

*Flowering Locus T* (*FT*) is the critical positive regulator of flowering in plants, and tobacco *FT* (*NtFt*) overexpression lines show an early flowering phenotype (Liu et al. 2019). We speculated that the Cas9-NtFT integrated protein could be cleaved by self-cleaving 2A peptide, such that Cas9 and NtFT5 could function in gene editing event and simultaneously triggering early flowering in dicots. The resulting expression system, pDC45_Fast, was constructed to test compatibility and editing efficiency in tobacco and tomato (Fig. 1A). Sequencing analysis showed editing efficiencies of 73.1%, 81.0% and 80.0%, respectively at *NtPDS, NtBRC1* and *NtCEN* alleles by the pDC45_Fast system, while the ho/bi mutation rates were 38.5%, 28.6%, and 45.0% for these genes. These results suggested that the pDC45_Fast construct could be used for relatively efficient genome editing in tobacco (Fig. 1P, Table S4). Additionally, editing of the *SlMYC2* site also showed relatively high efficiency in tomato (Fig. 1P, Table S4). Interesting, early bolting and fruit setting were observed in plants transformed with the pDC45_Fast system, and the time to first fruit setting was respectively shortened to 87 and 92 days for tomato and tobacco (Fig. 1O), compared to the normal regeneration process (around 120 and 150 days). To assess the inheritance of T0 homozygous mutants created by the pDC45_Fast system, we conducted a comprehensive analysis of the T1 progeny originating from line 5, derived from a *pDC45_Fast_BRC1*-edited plant. All eight T1 plants examined, which exhibited increased lateral branching, were confirmed to be homozygous for the same 1-bp deletion observed in the parental T0 line (Table S5), indicating that the homozygous mutations generated with the pDC45_Fast system were heritable. Additionally, we examined the T1 progeny for the presence of the vector based on the early-flowering phenotype, and found that approximately 25% of T1 lines that exhibited normal flowering time lacked the transgene elements, suggesting that the system could serve as an effective strategy for visual screening of transgene-free edited plants (Table S6). No editing events were detected at any of the tested potential off-target sites, implying that the pDC45_Fast system is highly specific in plants (Table S7). Although the pDC45_Fast system allows efficient editing and accelerates the generation of subsequent plant lines, we also observed some adverse phenotypic effects arising from this system during the regeneration process. Although regeneration itself was not significantly affected (Fig. S9), we encountered defects or complete loss of root formation in a notable number of regenerated tobacco shoots. In addition, some regenerated seedlings exhibited premature flowering in growth medium, consequently hindering seed production (Fig. S9). Specifically, no seeds were successfully harvested from regenerated pDC45_Fast tomato seedlings. Despite occasional loss of regenerated seedlings or failure to obtain seeds, the visual early-flowering phenotype allowed efficient selection of non-transgenic, edited plants at the T0 generation. This system can potentially serve as an effective tool for producing transgene-free plants and accelerating the molecular breeding process.

In summary, we identified a novel promoter, *PCE8pro*, that generates efficient gene editing in T0 plants. The pDC45 system is effective for multiplex gene editing and the deletion of chromosomal segments in dicot plants. The editing systems described here provide a new tool for improving targeted gene mutagenesis in dicots and will benefit future research in plants related to the functional analysis of gene families, the deletion of precursor miRNAs, and even large-scale genome editing.

## Supplementary Information

Supplementary Material 1.

Supplementary Material 2.

Supplementary Material 3.

Supplementary Material 4.

Supplementary Material 5.

## Data Availability

The datasets and materials that support the findings of this study can be obtained by contacting the corresponding author. The original data in this study are included in the paper and its supplementary materials.

## References

[CR1] Diamos AG, Mason HS. Chimeric 3’ flanking regions strongly enhance gene expression in plants. Plant Biotechnol J. 2018;16:1971–82. 29637682 10.1111/pbi.12931 PMC6230951

[CR2] Duan K, Cheng Y, Ji J, Wang C, Wei Y, Wang Y. Large chromosomal segment deletions by CRISPR/LbCpf1-mediated multiplex gene editing in soybean. J Integr Plant Biol. 2021;63:1620–31. 34331750 10.1111/jipb.13158

[CR3] Gao C. Genome engineering for crop improvement and future agriculture. Cell. 2021;184:1621–35. 33581057 10.1016/j.cell.2021.01.005

[CR4] Gao J, Wang G, Ma S, et al. CRISPR/Cas9-mediated targeted mutagenesis in Nicotiana tabacum. Plant Mol Biol. 2015;87:99–110. 25344637 10.1007/s11103-014-0263-0

[CR5] Guo Y, Ren G, Zhang K, Li Z, Miao Y, Guo H. Leaf senescence: progression, regulation, and application. Mol Hortic. 2021;1:5. 37789484 10.1186/s43897-021-00006-9 PMC10509828

[CR6] Hua K, Zhang J, Botella JR, et al. Perspectives on the application of genome-editing technologies in crop breeding. Mol Plant. 2019;12:1047–59. 31260812 10.1016/j.molp.2019.06.009

[CR7] Liu Y, Zeng J, Yuan C, et al. Cas9-PF, an early flowering and visual selection marker system, enhances the frequency of editing event occurrence and expedites the isolation of genome-edited and transgene-free plants. Plant Biotechnol J. 2019;17:1191–3. 30963647 10.1111/pbi.13118 PMC6577352

[CR8] Ma X, Zhu Q, Chen Y, Liu YG. CRISPR/Cas9 platforms for genome editing in plants: developments and applications. Mol Plant. 2016;9:961–74. 27108381 10.1016/j.molp.2016.04.009

[CR9] Ma Z, Ma L, Zhou J. Applications of CRISPR/Cas genome editing in economically important fruit crops: recent advances and future directions. Mol Hortic. 2023;3:1. 37789479 10.1186/s43897-023-00049-0 PMC10515014

[CR10] Tian Y, Chen K, Li X, Zheng Y, Chen F. Design of high-oleic tobacco (Nicotiana tabacum L.) seed oil by CRISPR-Cas9-mediated knockout of NtFAD2–2. BMC Plant Biol. 2020;20:233. 32450806 10.1186/s12870-020-02441-0 PMC7249356

[CR11] Xie K, Minkenberg B, Yang Y. Boosting CRISPR/Cas9 multiplex editing capability with the endogenous tRNA-processing system. Proc Natl Acad Sci U S A. 2015;112:3570–5. 25733849 10.1073/pnas.1420294112 PMC4371917

