# Supplementary material for: Efficient genome editing in dicot plants using calreticulin promoter-driven CRISPR/Cas system
Source: Mol Hortic. 2025 Feb 2;5:9. doi: 10.1186/s43897-024-00128-w (PMC11787731; doi:10.1186/s43897-024-00128-w)
Supplement: Supplementary file 1 — Supplementary Material 1. [file 43897_2024_128_MOESM1_ESM.pptx]

## Slide 1
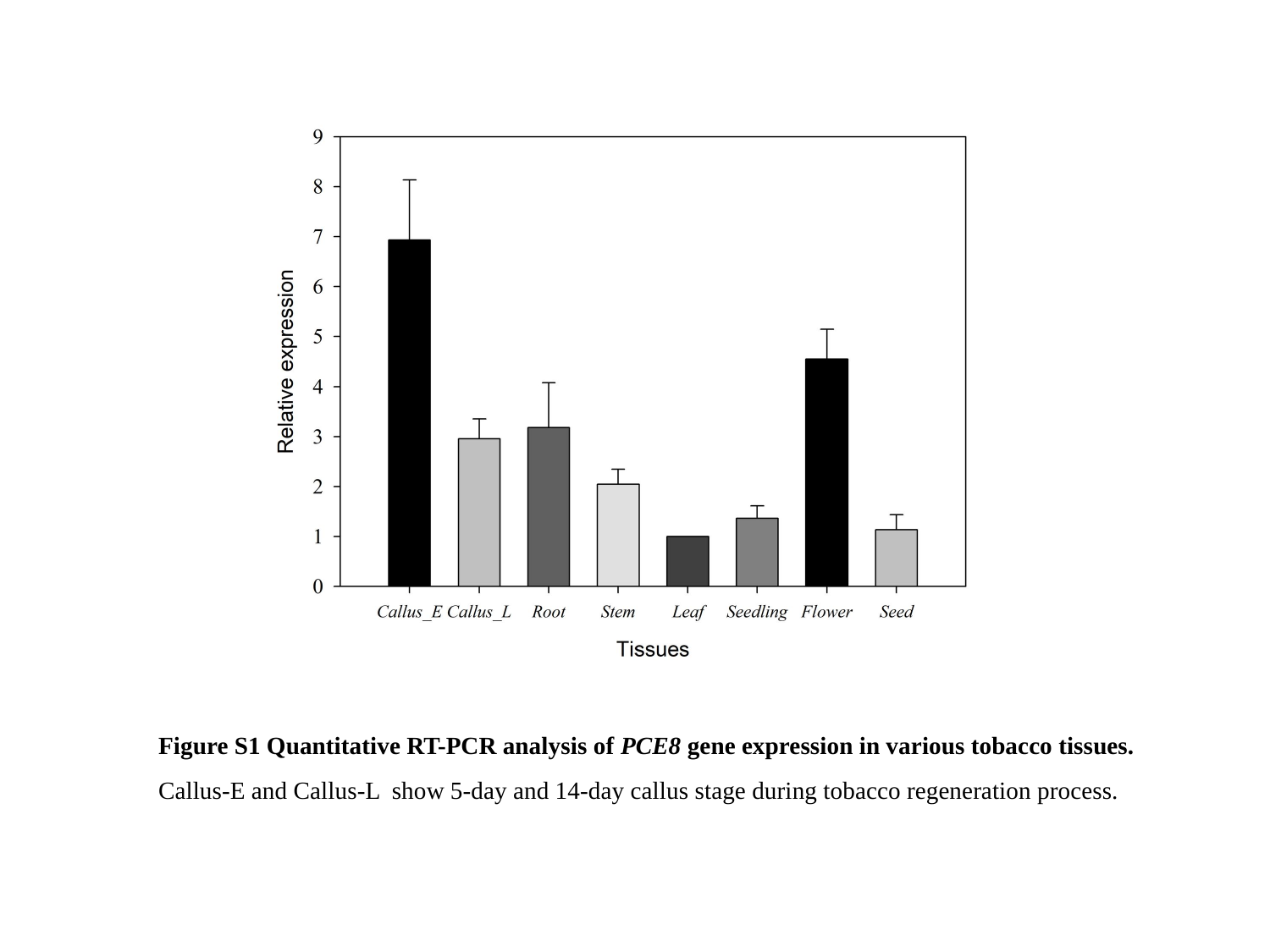

Figure S1 Quantitative RT-PCR analysis of PCE8 gene expression in various tobacco tissues.
Callus-E and Callus-L show 5-day and 14-day callus stage during tobacco regeneration process.

## Slide 2
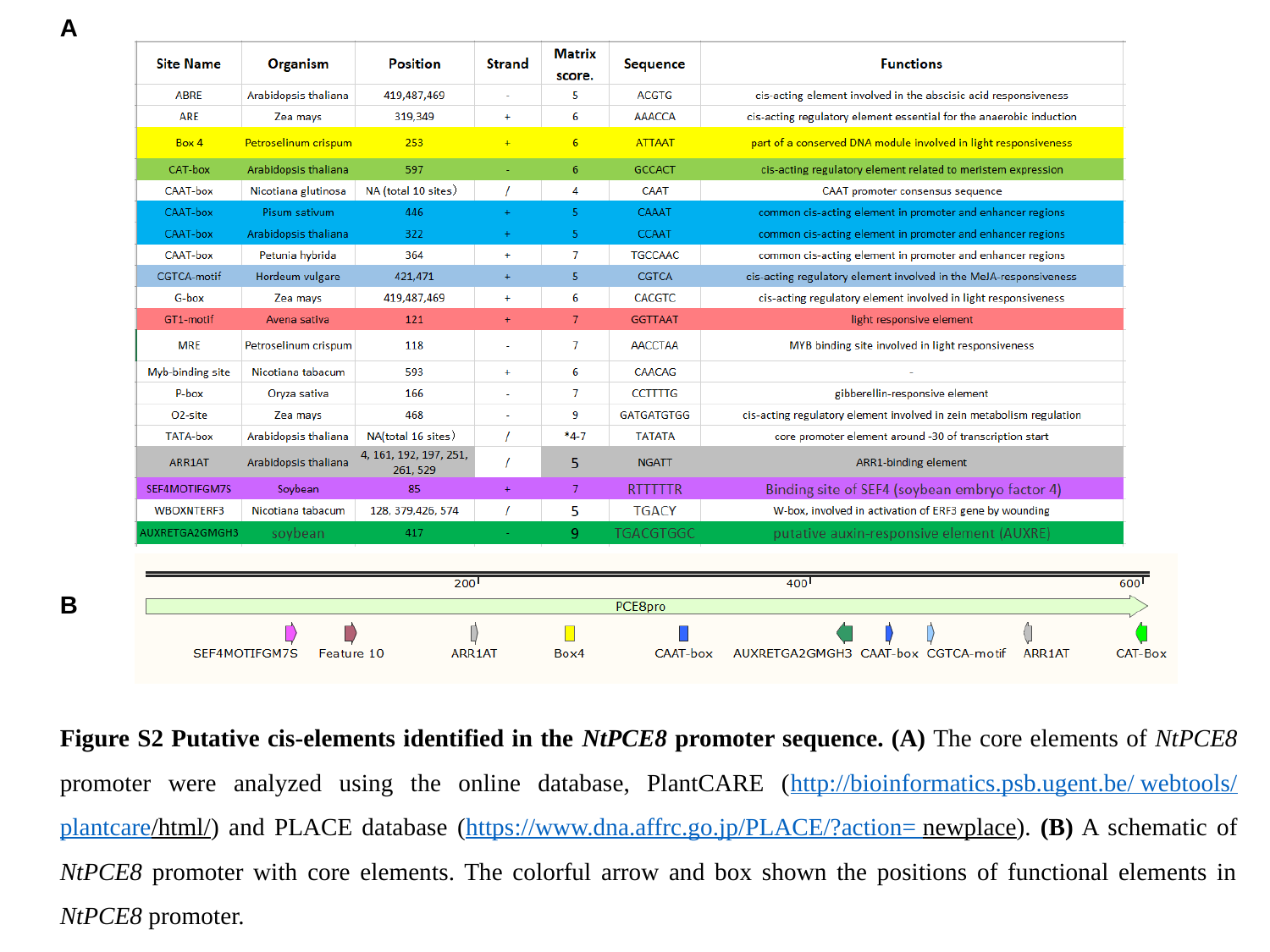

A
B
Figure S2 Putative cis-elements identified in the NtPCE8 promoter sequence. (A) The core elements of NtPCE8 promoter were analyzed using the online database, PlantCARE (http://bioinformatics.psb.ugent.be/ webtools/plantcare/html/) and PLACE database (https://www.dna.affrc.go.jp/PLACE/?action= newplace). (B) A schematic of NtPCE8 promoter with core elements. The colorful arrow and box shown the positions of functional elements in NtPCE8 promoter.

## Slide 3
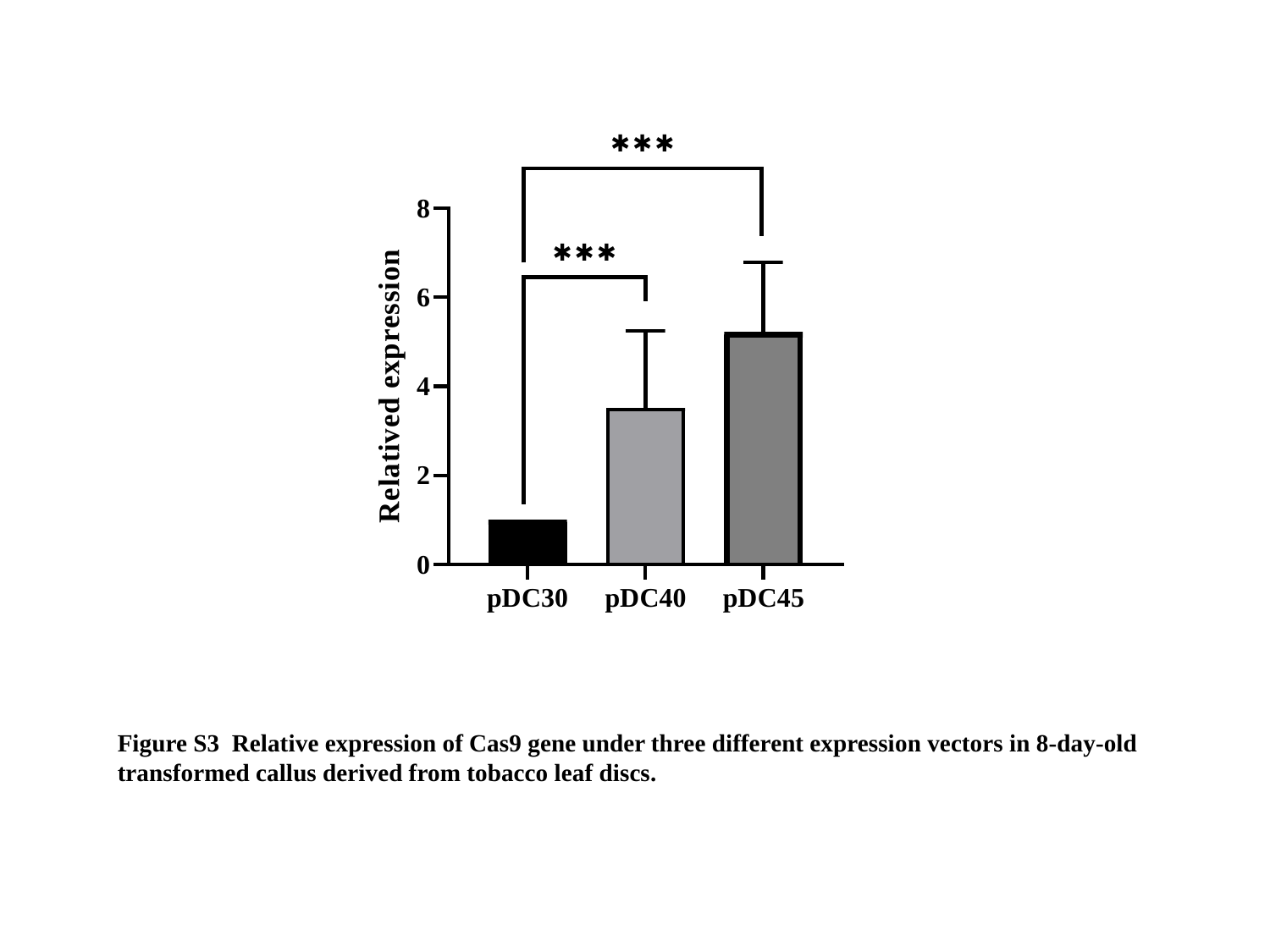

Figure S3 Relative expression of Cas9 gene under three different expression vectors in 8-day-old transformed callus derived from tobacco leaf discs.

## Slide 4
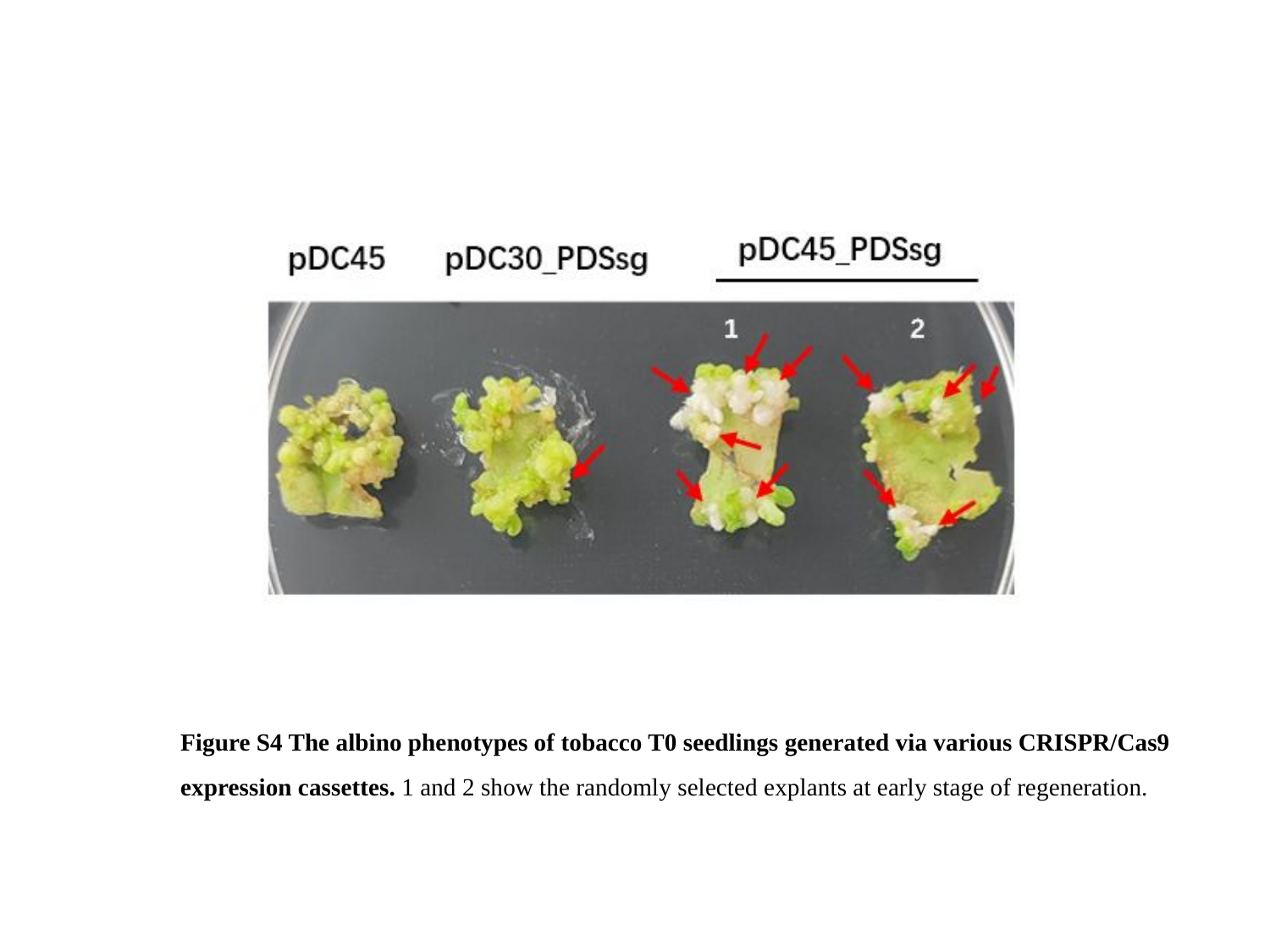

Figure S4 The albino phenotypes of tobacco T0 seedlings generated via various CRISPR/Cas9 expression cassettes. 1 and 2 show the randomly selected explants at early stage of regeneration.

## Slide 5
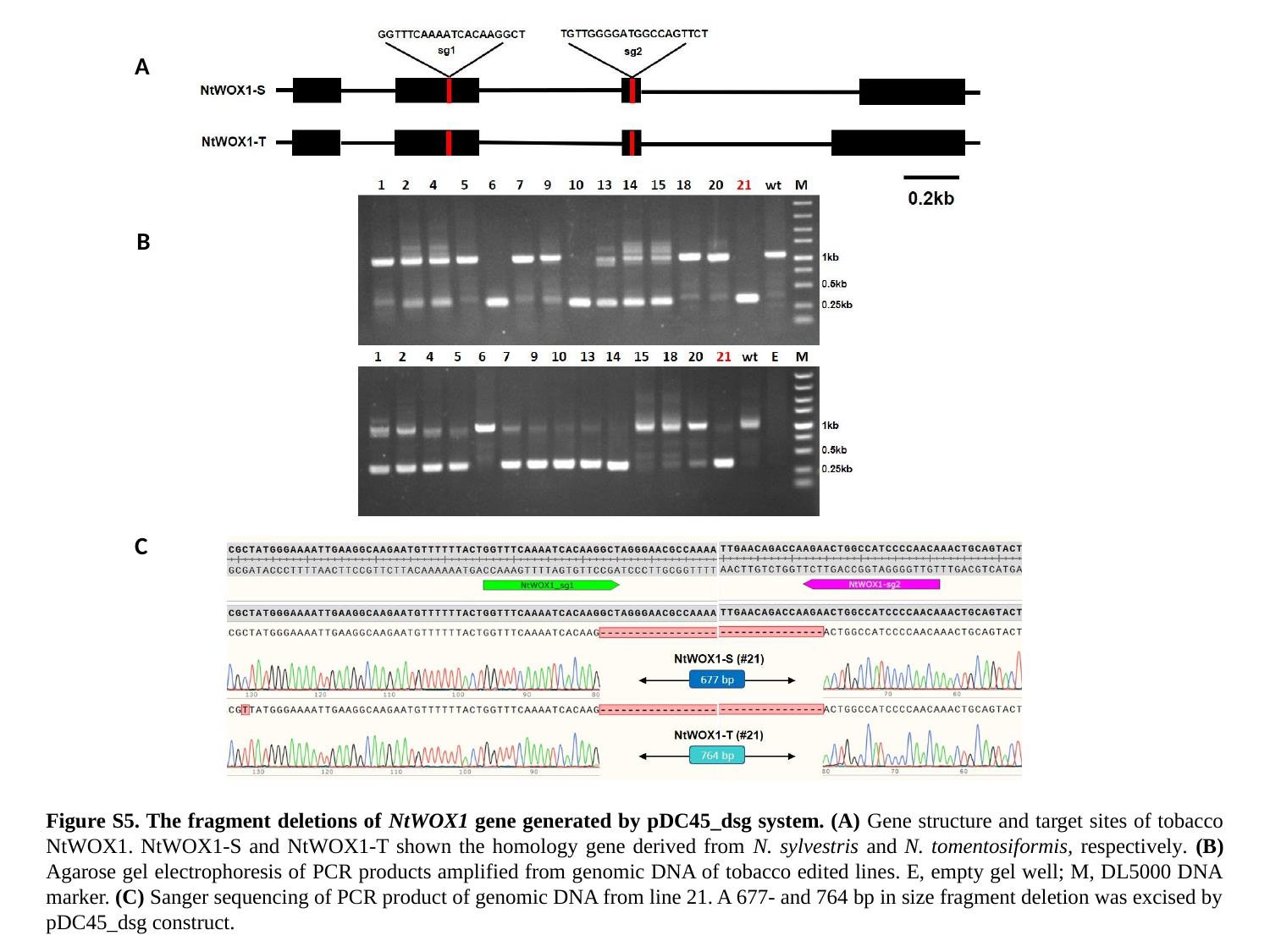

A
B
C
Figure S5. The fragment deletions of NtWOX1 gene generated by pDC45_dsg system. (A) Gene structure and target sites of tobacco NtWOX1. NtWOX1-S and NtWOX1-T shown the homology gene derived from N. sylvestris and N. tomentosiformis, respectively. (B) Agarose gel electrophoresis of PCR products amplified from genomic DNA of tobacco edited lines. E, empty gel well; M, DL5000 DNA marker. (C) Sanger sequencing of PCR product of genomic DNA from line 21. A 677- and 764 bp in size fragment deletion was excised by pDC45_dsg construct.

## Slide 6
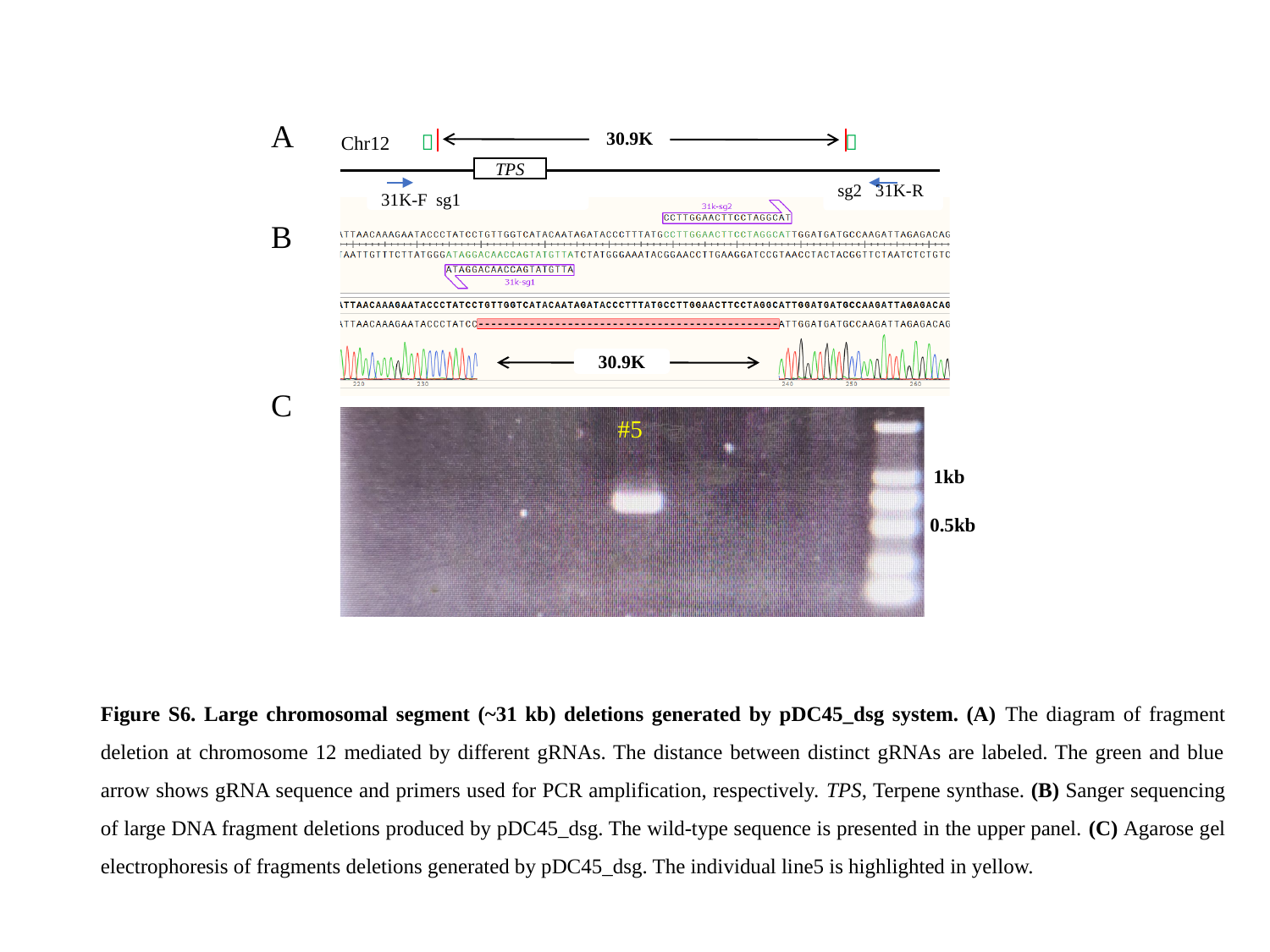

A


30.9K
Chr12
TPS
31K-F sg1
sg2 31K-R
30.9K
B
C
#5
1kb
0.5kb
Figure S6. Large chromosomal segment (~31 kb) deletions generated by pDC45_dsg system. (A) The diagram of fragment deletion at chromosome 12 mediated by different gRNAs. The distance between distinct gRNAs are labeled. The green and blue arrow shows gRNA sequence and primers used for PCR amplification, respectively. TPS, Terpene synthase. (B) Sanger sequencing of large DNA fragment deletions produced by pDC45_dsg. The wild-type sequence is presented in the upper panel. (C) Agarose gel electrophoresis of fragments deletions generated by pDC45_dsg. The individual line5 is highlighted in yellow.

## Slide 7
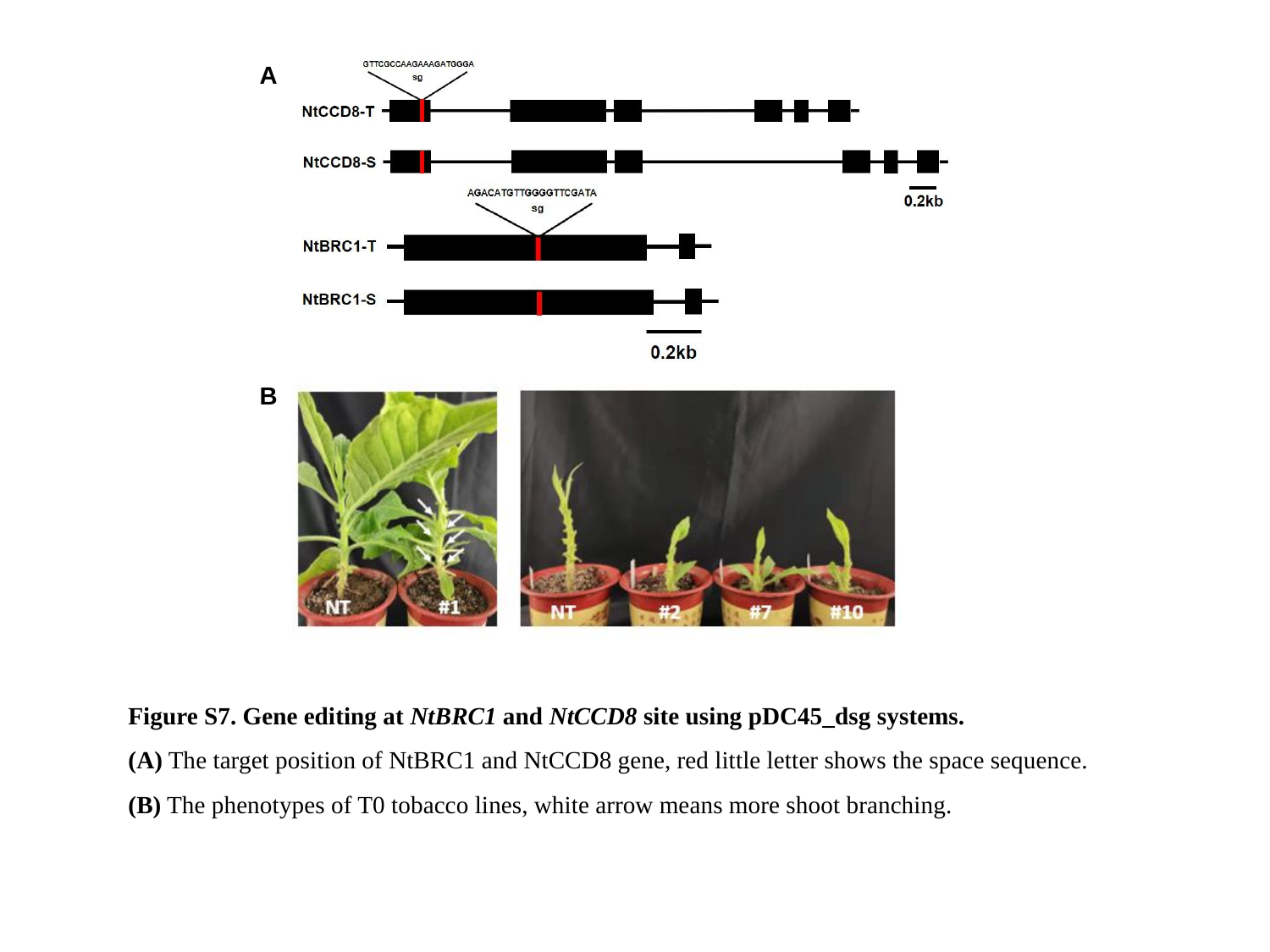

A
B
Figure S7. Gene editing at NtBRC1 and NtCCD8 site using pDC45_dsg systems.
(A) The target position of NtBRC1 and NtCCD8 gene, red little letter shows the space sequence.
(B) The phenotypes of T0 tobacco lines, white arrow means more shoot branching.

## Slide 8
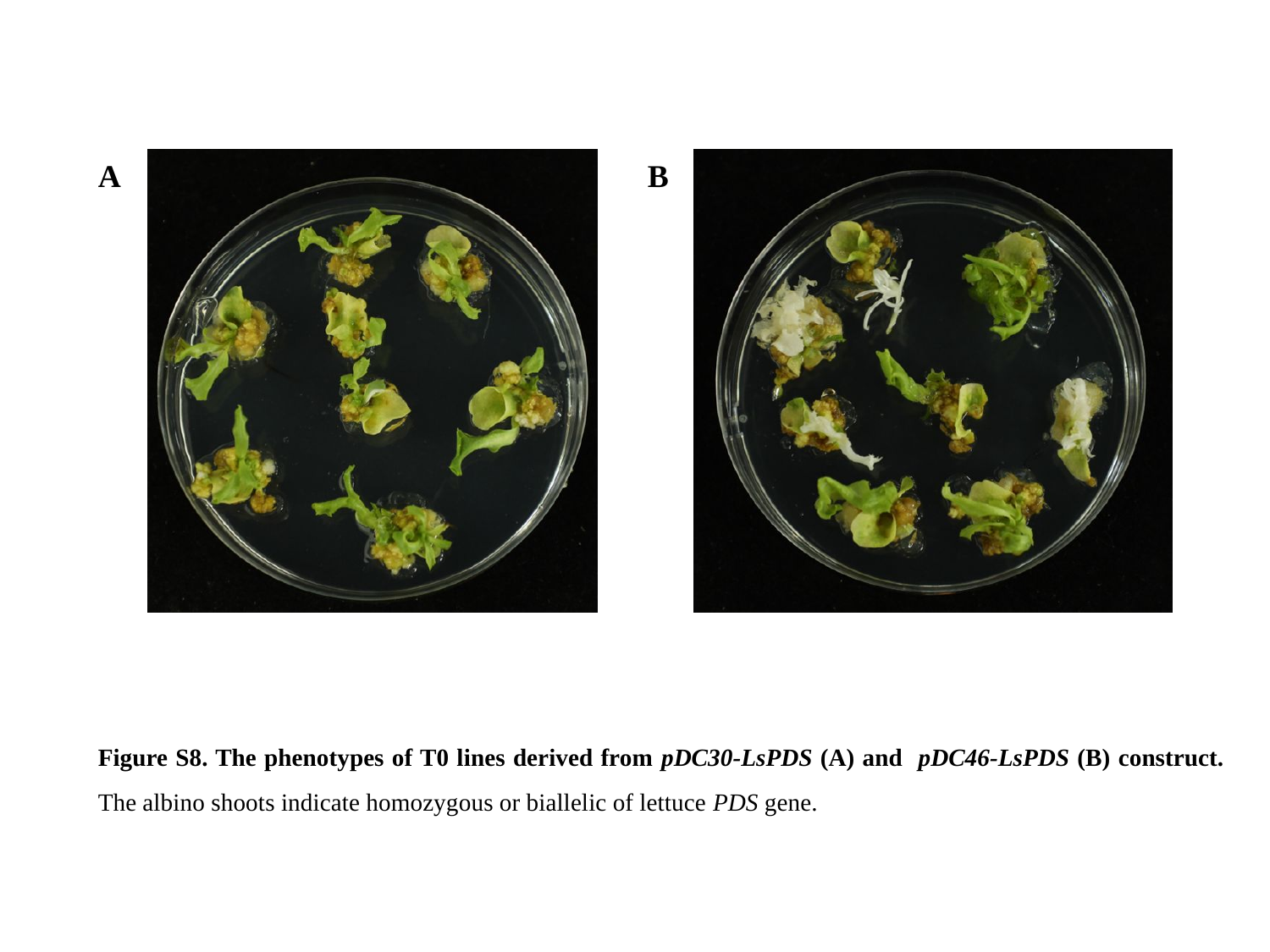

A
B
Figure S8. The phenotypes of T0 lines derived from pDC30-LsPDS (A) and pDC46-LsPDS (B) construct. The albino shoots indicate homozygous or biallelic of lettuce PDS gene.

## Slide 9
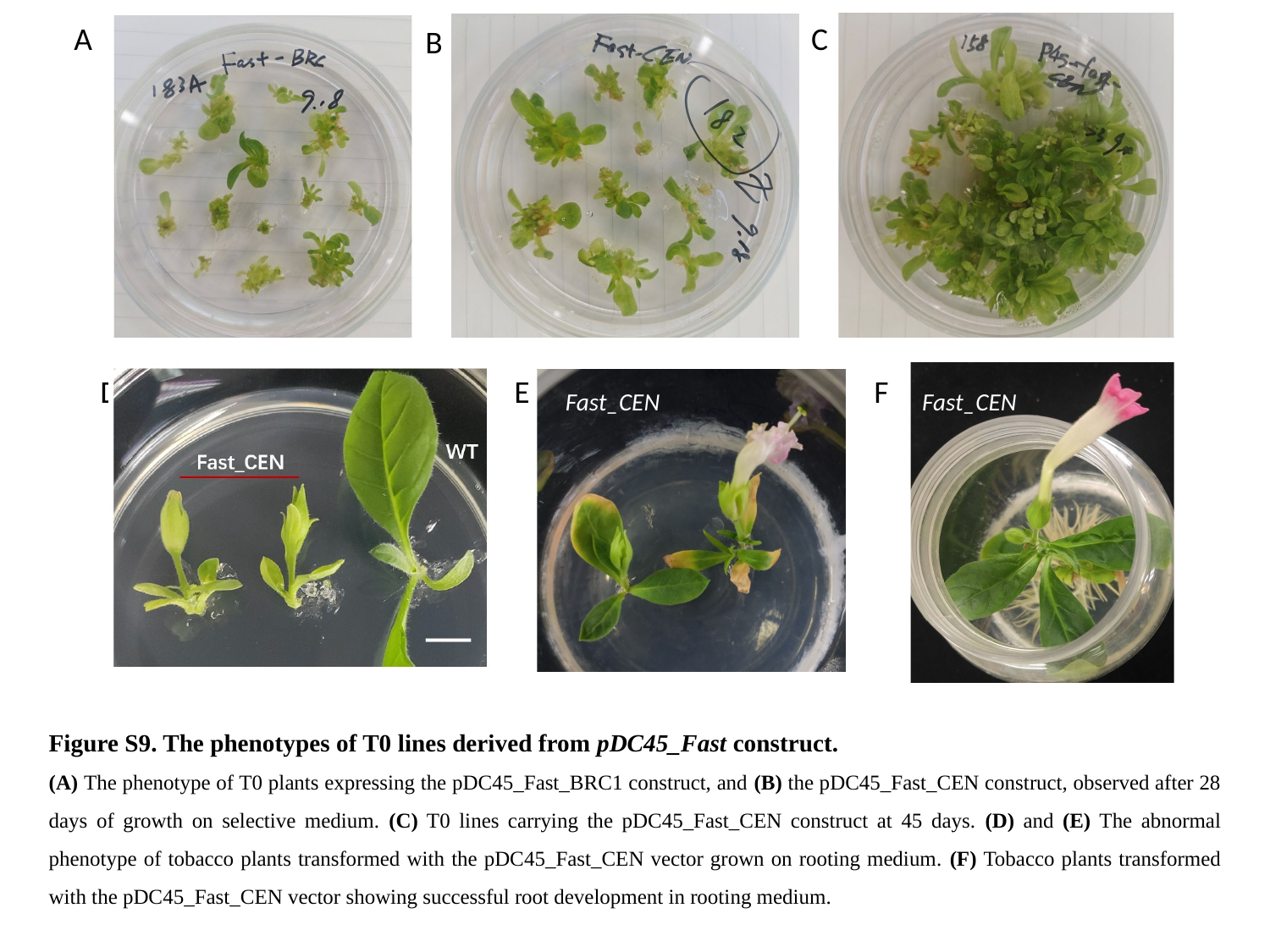

A
C
B
D E F
Fast_CEN
Fast_CEN
Figure S9. The phenotypes of T0 lines derived from pDC45_Fast construct.
(A) The phenotype of T0 plants expressing the pDC45_Fast_BRC1 construct, and (B) the pDC45_Fast_CEN construct, observed after 28 days of growth on selective medium. (C) T0 lines carrying the pDC45_Fast_CEN construct at 45 days. (D) and (E) The abnormal phenotype of tobacco plants transformed with the pDC45_Fast_CEN vector grown on rooting medium. (F) Tobacco plants transformed with the pDC45_Fast_CEN vector showing successful root development in rooting medium.
