# Supplementary material for: Efficient genome editing in dicot plants using calreticulin promoter-driven CRISPR/Cas system
Source: Mol Hortic. 2025 Feb 2;5:9. doi: 10.1186/s43897-024-00128-w (PMC11787731; doi:10.1186/s43897-024-00128-w)
Supplement: Supplementary file 3 — Supplementary Material 3. [file 43897_2024_128_MOESM3_ESM.docx]

**Supplemental material**

**The sequence of *PCE8* promoter**

AGTGG, cis-element related to meristem expression; GCCACGTCA, auxin-responsive element (AUXRE); TGACY, activation of ERF3 gene by wounding; CGTCA, MeJA-responsiveness

AATAGATTCATTGTTTACTTTTATTATGTATACACTATATAATTATTATACATGTACTATACTTATTTTATACCTCCAAAAGTTATTTTTAATTGAAGAGGTTGGGTTGACAACTATTTAGGTTAATTGACTTTTTAGGAATTCACCCTTTTTTAGCTTCAATCTCAAAAGGTAAATAGAAAAACAGAAAGAATAAAATCATTTTGATTGTACAAGTGCTAATTATTCTTTTAGTAAACAATAAATTCTAAGATTAATAGAATCGTTATATGCACGCCATTGTATGCGAAATTCATGTGAAAGAAAAAAGAGAGCAAAAAACCAATGGCTAAATTCGTTATTTTGAAAAAACCATCGCACAAGTGCCAACTTAATTGTGGTCACTTTCAACTCGTGGAGATACCCAACAAGCTTCAGCCACGTCAAGTCAATGACACTCTAATTACAAATGCCTCTTTTTAATTCGTCCACGTCATCGAACGTAGACACGTCGGACTAAGACCAAACGCTGCCCTACGTATCTACCAAAATCCTCTCGCCAGTATAAAAGAGCATTCATCAGAGATCTTTTAGAGTCAGTATTTCGATCTCACAACAGTGG

**The sequence of LsPCE8 promoter from lettuce**

TTTATTATAAATTTGATCTGAAATTACTATTATACCTTTTTTTCAATTTATTTTCCTTTTTTAATATAATTTTGCTTTAAAGAAAGAAAAAAAACCCACCCCACCCCACTTTACCACCACCCTTTATTTGCGTTTACATGATTTTACTCCCAAGTCCCACCCACCACCGGACTCCTTGCATGTTGTTGATTCTCTCTTTTTCTCTATATTTATTCTCTCGTTCTCCTGTTTATCTCGAAAGGAAAAAAATAGAAGAAGGCCATCGCTTCTTCCTGCTCCCCCAACCCCTAACATTGTAATATTTGTTTTCTTAACAAACACTACCCTAAGAGATATAGATCGTTGTGAGATGAACAGATGAGATAAAGCCTTAACTTGCTTTAATTTGAGATGTGTTATTTAATAAAATATAGTTGAGAAATGTTGTAGTTCGTGTGATAAGGATTTCGTGGATATAAAGTTTGTTGAAAATGGAGTTTGTATGAGAAAGTTACGACTATTTGAAGAGATGAAAGTATGTTGTTATTTTGAGAAAGTCAACACAGGTCAAATTAAAGGGTCAATCTTTGACTACCACAACGTGAACAATAGATCATCACGGCATGACTAATAGTCATCAAGACGTGACATATGACGAGTCACCGAGACACGTGGCAAAATCGGGGTTCAATTGTTCATCACGATGTGACACATATATGGTCATGACGTGACCATTGCAAGTTATAAATATGCAAGTTTTTGTGTTATTTGGGGGATTTTTGGCACATTCTATTGGAGAAATGTGTGCATCAAAAAACATGTCGTGCTGCACAAATAAAATGTGTTATCATTTCATCCCAAAACGAAAACTGACAAATATTCTGAACTAGTTCCTCGTGTTCATGACGAGTTCCTCGTGTTCCGGAACAGTTCCTCGCATTCTGGAACAGTTCCTCGGAGTTGTGTGTCGGAGATCAGAATTAGGATCGGGGTCGCGTGTCGGAATCGAATCGGAAAGTCGGAGCAAGATCTGGAATGAAGGCAAGGGTCTGAATTTTTTCAAATTATATTACAGAATTTGAATATCAAATTCCCCATCCAGAACACGAACCGATAGTCCGTAATCAGAATTTTGGAGGCACGGTAATGTGCCGACCCACCCGAAAATGAGTCTGAAATACTCAAATTCCAAAATAAATGATTATTTAAAAAAAATTATAATTTTAAAAAACACACAAAAAAAATATTAGATTACTCGATTTACCTTTTAACATAAAATACGGTTTTATTGATGGGCATCAATATTCTCAATAAATGAATAAACATAGGGTTAACCTGGTAATTTGGCATTTTCATAAACCCATATATCTACATGGCAAGATCGTTGGATCACCCAATCGACATCAGCCACGTTGCACTTTATGACAAACTGGTCTGACATGGCTAGCTTTAATTCGTCCACGTCACTCTTCCCTCCACTGATACAAACGTAACGACCATAAGACGACCTCCTTATAAACCCTACTATCACTTTCTTGCTCTAGCGGCGGGATCTCACTTTCTACTCTCTGGTC

**AtU6-sgRNA**

Light green, AtU6-26; Yellow, sgRNA scanfold

AAGCTTACTAGTCATTCGGAGTTTTTGTATCTTGTTTCATAGTTTGTCCCAGGATTAGAATGATTAGGCATCGAACCTTCAAGAATTTGATTGAATAAAACATCTTCATTCTTAAGATATGAAGATAATCTTCAAAAGGCCCCTGGGAATCTGAAAGAAGAGAAGCAGGCCCATTTATATGGGAAAGAACAATAGTATTTCTTATATAGGCCCATTTAAGTTGAAAACAATCTTCAAAAGTCCCACATCGCTTAGATAAGAAAACGAAGCTGAGTTTATATACAGCTAGAGTCGAAGTAGTGATTAGAGACCGAGGTCTCGGTTTTAGAGCTAGAAATAGCAAGTTAAAATAAGGCTAGTCCGTTATCAACTTGAAAAAGTGGCACCGAGTCGGTGCTTTTTTGTTTTAGAGCTAGAAATAGCAAGTTAAAATAAGGCTAGTCCGTAGCGCGTGCGCCAATTCTGCAGACAAATGCTAGCCTGCAGGCCCCGGGcccagGGATCC

**tRNA-BsaI_sg**

Green, tRNA; Yellow, sgRNA scaffold

AACAAAGCACCAGTGGTCTAGTGGTAGAATAGTACCCTGCCACGGTACAGACCCGGGTTCGATTCCCGGCTGGTGCAAGAGACCGAGGTCTCGGTTTTAGAGCTAGAAATAGCAAGTTAAAATAAGGCTAGTCCGTTATCAACTTGAAAAAGTGGCACCGAGTCGGTGCAACAAAGCACCAGTGGTCTAGTGGTAGAATAGTACCCTGCCACGGTACAGACCCGGGTTCGATTCCCGGCTGGTGCA
