# Supplementary material for: Efficient genome editing in dicot plants using calreticulin promoter-driven CRISPR/Cas system
Source: Mol Hortic. 2025 Feb 2;5:9. doi: 10.1186/s43897-024-00128-w (PMC11787731; doi:10.1186/s43897-024-00128-w)
Supplement: Supplementary file 4 — Supplementary Material 4. [file 43897_2024_128_MOESM4_ESM.docx]

**SUPPORTING INFORMATION**

**Supplementary methods**

The primers used in this work are listed in Supplementary Table 8. The sequence of *PCE8pro*, *AtU6-sgRNA*, and *tRNA-BsaI_sg* is listed in the Supplemental material.

**Plant material and sample preparation**

The cultivated tobacco (*Nicotiana. tabacum*), the lettuce (*Lactuca sativa*), and the Micro-Tom (*Solanum lycopersicum*) were grown in the greenhouse. The genomic DNAs of tobacco and tomato were extracted using the methods described previously (Lv et al. 2021a).

**Vector construction**

To construct the p1300-U6_sg, the synthetic AtU6-sgRNA fragment (Supplemental material) was inserted into the *HindIII-EcoRI* site of modified pCAMBIA1300 binary vector, with *BsaI* and *NheI* cloning sites disrupted. To increase the stability of the *Cas9* gene in plants, the Cas9-PA50 fragment was amplified using *Cas9-NcoI-F* and *Cas9-PA50-R* primers, then cloned into the *NcoI-BamHI* site of 18T-U6-sg-Cas9 vector (Zhang et al. 2016), resulting in the generation of the 18T-U6-sg-Cas9-PA50 vector. The AtUBQ1pro-Cas9-PA50 was subsequently introduced into the *HindIII-EcoRI* site of p1300-U6_sg vector, producing the pDC01 binary vector.

To construct the pDC30 and pDC40 vectors, we replaced the AtUBQ1pro fragment with 35Spro and PCE8pro, respectively, at the XmaI-NcoI site of the pDC01 vector. To obtain the pol II promoter-driven *tRNA-sgRNA* unit, the *2X35Spro* and *EU* terminator (Rosenthal et al. 2018; Diamos and Mason 2018) were amplified with corresponding primers (Table S4), the *tRNA-BsaI_sg* was synthesized from BGI Tech (BEIJING LIUHE). Three fragments were integrated by overlap PCR method, generating the 35Spro-tRNA_sgRNA-EU product. The 35Spro-tRNA_sgRNA-EU fragment with an adaptor, was amplified with *35S-CE-F* and *EU-CE-R* primer, and introduced into the *SpeI-SbfI* site of pDC40 via homologous recombination method, producing the pDC45 vector. To generate the pDC45-LsPCE8pro construct (designated as pDC46), a 1.5 kb promoter region upstream of *LsPCE8* the lettuce ortholog of *PCE8*, was amplified from lettuce gDNA using LsPCE8pro-hrF and LsPCE8pro-hrR primers (Supplementary Table 8). We then digested pDC45 with the *SbfI* and *NcoI* restriction enzymes, and replaced *NtPCE8* promoter with *LsPCE8pro* for driving the *Cas9* expression using the ClonExpress Ultra One step cloning kit V3 (C117-02, Vazyme). The gRNAs used in this study were synthesized and inserted at *BsaI* site of pDC expressing vectors using the T4 ligase (2011A, Takara). For the dual sgRNA system, the fragment was amplified with the corresponding primers, and subsequently introduced into pDC45_dsg vector using Golden Gate Assembly Kit (E1601L, New England Biolabs). The tobacco *FT* gene was amplified with *NtFT-F* and *NtFT-R* primers, the *P2A-FT-F* and *NtFT-BamHI-CE-R* were used to generate FT-overlap fragment, and the Cas9-2A product was obtained with *CAS9-NcoI-CE-F* and *Cas9-linker-R* primer with pDC45 vector as a template. The Cas9-P2A-FT product with an adaptor, was generated by overlap PCR, and subsequently cloned into *NcoI-BamHI* site of pDC45 vector, resulting in the generation of pDC45_Fast. All constructions were confirmed by Sanger sequencing.

**Plants transformation**

The stable transformation of tobacco was performed as previously described, with minor modifications (Lv et al. 2021a; Lv et al. 2021b). Briefly, *Agrobacterium tumefaciens* strain *EHA105*, harboring various constructs, were cultured in LB medium with rifampicin and kanamycin at 28°C. Upon reaching OD600 cell densities of 0.6 - 0.8, cells were pelleted and resuspended in MS medium with 30 g/L sucrose and 20 mg/L acetosyringone. Tobacco leaf discs were immersed in bacterial suspension for 8–10 minutes, then incubated in antibiotic-free MS medium in darkness for 3 days, and subsequently transferred to a differentiation medium (MS, 2.5 mg/L 6-benzylaminopurine, 0.2 mg/L NAA, 10 mg/L hygromycin, hygromycin, 150 mg/L timentin) to promote shoot formation. Regenerated plantlets were moved to rooting medium (MS with 0.1 mg/L NAA and 200 mg/L cefotaxime). Resistant seedlings were then transplanted in soil for further analysis

The protocol for lettuce transformation was previously described (Pan et al. 2022). Briefly, the leaf discs of 5-day-old seedlings obtained from sterilized lettuce seeds were incubated with recombinant Agrobacterium (*EHA105*) for 15min in half-strength MS media MS media (30 g L^–1^ sucrose, 2.23g L^-1^ MS salt, 200 uM acetosyringone, pH 5.8) followed by the co-cultivation on the MS media in the dark at 25℃ for 48h. Subsequently, the leaf discs were transferred to MS selective media (30 g L^–1^ sucrose, 2.23g L^-1^ MS salt, pH 5.8) with 2mg L^-1^ hygromycin. Regenerated shoots were excised after 4 weeks and grown on rooting media (1/2 MS supplemented with 15 g L^–1^ sucrose, 2 mg L^–1^ hygromycin) for root induction. The plantlets with well-developed shoot and root were transferred to soil and further confirmed. Transformation of transgenic tomato was conducted using the Agrobacterium-mediated cotyledon transformation method as described previously (Van Eck et al. 2019).

**qRT-PCR assays**

Total RNA was extracted from 8-day-old calli of leaf discs transformed with the pDC30, pDC40, or pDC45 vectors using FreeZol Reagent (Vazyme, Nanjing, China). The corresponding cDNA was synthesized using SYBR Green Premix Pro Taq with the qHS qPCR Kit (Accurate Biotechnology, Hunan, China) according to the manufacturer's instructions. Expression levels of *Cas9* in tobacco callus tissue were determined by qRT-PCR, with the *NtUBC* gene (Nitab4.5_0008519g0010) serving as an internal control. Candidate gene expression levels were quantified using the 2^−ΔΔCt^ method. The primers used for qRT-PCR assays are listed in Supplementary Table 8.

**In silico analysis**

Amino acids sequences of plant calreticulin-like proteins (CRT) proteins were retrieved from the NCBI database using the tobacco PCE8 sequence as a reference. The tobacco CRT gene family was aligned using DNAMAN software. Amino acids sequences from different species were aligned using ClustalW2 (http://www.ebi.ac.uk/Tools/clustalw2/). Phylogenetic trees, based on the resultant alignments, were constructed with MEGA 6.0 software using the Neighbor-Joining tree method (Tamura et al. 2013). The sequence ID used for alignment and phylogenetic tree construction showed as follows: Cs, *Cucumis* *sativus* (XP_004144757.1); Mt *Medicago truncatula* (XP_024638651); Ah, *Arachis hypogaea* (XP_025674985); Gm, *Glycine* *max* (KAG5077294.1); Pt, *Populus* *trichocarpa* (XP_002318957.1); Ls *Lactuca* *sativa* (RVX01449); Gh, *Gossypium* *hirsutum* (XP_016667890.2); At, *Arabidopsis* *thaliana* (NP_176030.1) ; Bn, *Brassica* *napus* (XP_013687715); Ca, *Capsicum* *annuum* (PHT61563.1); St, *Solanum* *tuberosum* (KAH0766737.1); Sl, *Solanum* *lycopersicum* (XP_004230299.1); Zm, *Zea* *mays* (ONM52621.1); Os, *Oryza* *sativa* (KAF2922127); Ta, *Triticum* *aestivum* (XP_044391420.1); Hv, *Hordeum* *vulgare* (XP_044949696); Pv, *Panicum* *virgatum* (XP_039830141); Sb, *Sorghum* *bicolor* (XP_021307591).

**Transgenic events and mutation detection**

Genomic DNA from the tobacco regenerated lines was extracted using either the Plant Direct PCR Kit (PD105-02, Vazyme) or the NuClean Plant Genomic DNA Kit (CW0531M, CWBIO), following the relevant guidelines. T0 plants were further confirmed by amplification of SpCas9 using SpCas9-1043F and SpCas9-1590R primers (Supplementary Table 8). The T1 progeny of the T0 lines generated by *pDC45_Fast* were grown in pots, and genomic DNA was extracted from the leaves of 45-day-old T1 plants. The presence of transgenic elements was verified by amplifying the *SpCas9* gene. To detect the targeted deletion and mutation of pDC45_PDSsg and pDC45_WOX1sg transformed lines, the PCR product amplified with PDS-del and WOX1-del primers (Table S4), were either directly sequenced or ligated to the pEASY-Blunt Zero vector, and introduced into *E.coli* (Transgene, China). Ten or more of the colonies were sequenced to analyze the mutation types. To analyze the editing efficiency of diverse pDC systems in T0 or T1 plants, the corresponding adaptor primers were used to amplify target fragments for deep sequencing. The data were collected and analyzed using the Hi-TOM platform (Liu et al. 2019).

**Off-target analysis**

The potential off-target sites with 2 to 4 nucleotide mismatches compared to the sgRNAs of *NtBRC1* and *SlMYC2* were searched using Cas-OFFinder tool (Bae et al. 2014). Fifteen mutant lines were randomly selected, and the PCR products from target regions of each plants were amplified using the corresponding primer for Hi-TOM sequencing.

**Statistical analysis**

The representative values are presented as means ± standard deviation (SD) derived from at least three replicates. Statistical significance between control and the treatments were analyzed by Student’s *t*-tests using SPSS software (**P* < 0.05, ***P* < 0.01, ****P* < 0.0001). ns means no significant difference.

**References:**

Bae S, Park J, Kim JS (2014) Cas-OFFinder: a fast and versatile algorithm that searches for potential off-target sites of Cas9 RNA-guided endonucleases. Bioinformatics 30:1473-1475.

Diamos AG, Mason HS (2018) Chimeric 3' flanking regions strongly enhance gene expression in plants. Plant Biotechnol J 16:1971-1982.

Liu Q, Wang C, Jiao X, Zhang H, Song L, Li Y, Gao C, Wang K (2019) Hi-TOM: a platform for high-throughput tracking of mutations induced by CRISPR/Cas systems. Sci China Life Sci 62:1-7.

Lv J, Chen YQ, Ding AM, Lei B, Yu J, Gao XM, Dai CB, Sun YH (2021a) Control of axillary bud growth in tobacco through toxin gene expression system. Sci Rep 11:17513.

Lv J, Dai CB, Wang WF, Sun YH (2021b) Genome-wide identification of the tobacco GDSL family and apical meristem-specific expression conferred by the GDSL promoter. BMC Plant Biol 21:501.

Pan W, Liu X, Li D, Zhang H (2022) Establishment of an Efficient Genome Editing System in Lettuce Without Sacrificing Specificity. Front Plant Sci 13:930592.

Rosenthal SH, Diamos AG, Mason HS (2018) An intronless form of the tobacco extensin gene terminator strongly enhances transient gene expression in plant leaves. Plant Mol Biol 96:429-443.

Tamura K, Stecher G, Peterson D, Filipski A, Kumar S (2013) MEGA6: Molecular Evolutionary Genetics Analysis version 6.0. Mol Biol Evol 30:2725-2729.

Van Eck J, Keen P, Tjahjadi M (2019) Agrobacterium tumefaciens-Mediated Transformation of Tomato. Methods Mol Biol 1864:225-234.

Zhang Z, Mao Y, Ha S, Liu W, Botella JR, Zhu JK (2016) A multiplex CRISPR/Cas9 platform for fast and efficient editing of multiple genes in Arabidopsis. Plant Cell Rep 35:1519-1533.
