# Supplementary material for: Efficient genome editing in dicot plants using calreticulin promoter-driven CRISPR/Cas system
Source: Mol Hortic. 2025 Feb 2;5:9. doi: 10.1186/s43897-024-00128-w (PMC11787731; doi:10.1186/s43897-024-00128-w)
Supplement: Supplementary file 5 — Supplementary Material 5. [file 43897_2024_128_MOESM5_ESM.docx]

Abbreviations

CRT Calreticulin-like protein

NtPDS PHYTOENE DESATURASE

NtCIPK Calcineurin B-like protein (CBL) interacting protein kinase

NtWOX1 WUSCHEL related homeobox 1-like genes

NtBRC1 BRANCHED 1

NtCEN CENTRORADIALIS

NtCLC Chloride channel

NtPPD PEAPOD
